# Personality, Healthcare Use and Costs—A Systematic Review

**DOI:** 10.3390/healthcare8030329

**Published:** 2020-09-09

**Authors:** André Hajek, Benedikt Kretzler, Hans-Helmut König

**Affiliations:** Department of Health Economics and Health Services Research, University Medical Center Hamburg-Eppendorf, 20246 Hamburg, Germany; b.kretzler.ext@uke.de (B.K.); h.koenig@uke.de (H.-H.K.)

**Keywords:** big five, GP visits, healthcare use, health services research, neuroticism, personality, primary care, systematic review

## Abstract

Background: Thus far, there is a lack of a systematic review synthesizing empirical studies that analyze the link between personality factors and healthcare use (HCU) or costs. Consequently, the purpose of our systematic review is to give an overview of empirical findings from observational studies examining the association between personality factors and HCU or costs. Methods: PubMed, PsycINFO, and NHS EED (NHS Economic Evaluation Database) were searched. Observational studies examining the association between personality factors and HCU costs by using validated tools were included. Two reviewers performed study selection and data extraction and evaluated the study quality. Findings were synthesized qualitatively. Results: In total, n = 15 studies (HCU, n = 14; cost studies, n = 1) were included in the final synthesis. A few studies point to an association between conscientiousness and HCU (with mixed evidence). Some more evidence was found for an association between higher agreeableness, higher extraversion, and higher openness to experience and increased HCU. The majority of studies analyzed found a link between higher neuroticism and increased HCU. Conclusion: Personality factors, and particularly neuroticism, are associated with HCU. This knowledge is important to manage healthcare use. However, future research based on longitudinal data and studies investigating the link between personality characteristics and costs are required.

## 1. Introduction

Knowledge about the factors associated with healthcare use (HCU) is key to manage healthcare resources and, therefore, to avoid overuse or misuse. Commonly based on the Andersen Behavioral Model [1], various studies have examined the determinants of HCU [2,3]. According to this model, it is possible to distinguish between predisposing characteristics such as age or sex, enabling resources such as access to doctors or income, and need factors such as somatic morbidity or self-rated health. Existing systematic reviews have shown that HCU is largely driven by need factors [4,5]. However, some recent cross-sectional and longitudinal studies have demonstrated that personality factors are also linked to HCU [6,7]. Friedman and colleagues [6] demonstrated, among others, a link between neuroticism and HCU in the United States using a cross-sectional approach. Another longitudinal study from Germany [7], showed that while an intraindividual increase in neuroticism was associated with an intraindividual increases in physician visits, an increase in extraversion was associated with an increased probability of hospitalization.

While we acknowledge the fact that other models of personality structure exist (e.g., HEXACO (Honesty-Humility, Emotionality, Extraversion, Agreeableness, Conscientiousness, and Openness to Experience) [8]), personality is most commonly divided into five traits (also called “big five”) [9]: (1) agreeableness (for example, to get along well with others), (2) conscientiousness (degree to which an individual is persevering, reliable, and careful), (3) extraversion (e.g., have a positive outlook in life and to experience positive emotions), (4) neuroticism (e.g., experience negative emotions such as anger or anxiety), and (5) openness to experience (e.g., to be imaginative or open-minded).

Thus far, there is a lack of studies systematically synthesizing studies that examine the link between personality factors and HCU or costs. Therefore, the objective of our systematic review is to provide an overview of evidence examining this association. This knowledge is important for managing healthcare use.

## 2. Materials and Methods

In accordance with the Preferred Reporting Items for Systematic Reviews and Meta-Analysis Protocols guidelines [10], our systematic review was conducted. Our systematic review is registered to the International Prospective Register of Systematic Reviews (PROSPERO, registration number: CRD42020170800).

### 2.1. Search Strategy and Selection Criteria

In three databases (PubMed; PsycINFO, and NHS EED), a systematic literature search was performed in March 2020. The search query for PubMed is given in Table 1.

Studies were evaluated for inclusion/exclusion using a two-step process, which was independently performed by two reviewers (A.H., B.K.) using defined selection criteria: (1) title/abstract screening and (2) full-text screening. Furthermore, the two reviewers investigated the reference lists of the articles included in our review to identify articles that could be important. If disagreements occurred, a consensus was reached through discussion or by inclusion of a third party (H.-H.K.).

The inclusion criteria were as follows: (i) observational studies (cross-sectional and longitudinal) analyzing the link between personality factors and HCU or costs in all age categories, (ii) studies using validated tools to quantify personality factors, (iii) publications in English or German language published in peer-reviewed journals. The exclusion criteria were as follows: (i) studies that do not report the link between personality factors and HCU or costs, (ii) studies only focusing on mental HCU, (iii) studies exclusively investigating samples with a specific disorder such as individuals with personality disorders, (iv) study design other than observational, (v) measurement of personality or HCU or costs not appropriate, (vi) studies not published in peer-reviewed journals or in languages other than German or English. No restrictions were applied regarding location or time of the publication. Using a sample of 100 titles/abstracts, a pretest was conducted prior to final eligibility criteria. However, eligibility criteria remained the same.

### 2.2. Data Extraction and Analysis

One reviewer (A.H.) performed the data extraction and a second reviewer (B.K.) cross-checked it. Disagreements were resolved through discussion, by inclusion of a third party (H.-H.K.), or by contacting the authors of the study.

Data on the design of the study, definition and measurement of important variables, sample characteristics, statistical analysis, and key findings regarding the association between personality factors and HCU or costs were extracted. In the results section, the main findings are presented for each personality factor separately.

### 2.3. Quality Assessment

There is no consensus on a quality assessment measurement for both HCU and cost studies. Therefore, we adapted previous checklists for HCU and cost studies developed by Stuhldreher et al. [11] and refined by Hohls et al. [12]. The quality assessment was performed by two reviewers (A.H., B.K.). Disagreements were resolved through discussion or, if required, through inclusion of a third party (H.-H.K.). The results of the quality assessment are displayed in the results section.

## 3. Results

### 3.1. Overview: Included Studies

**Figure 1:**
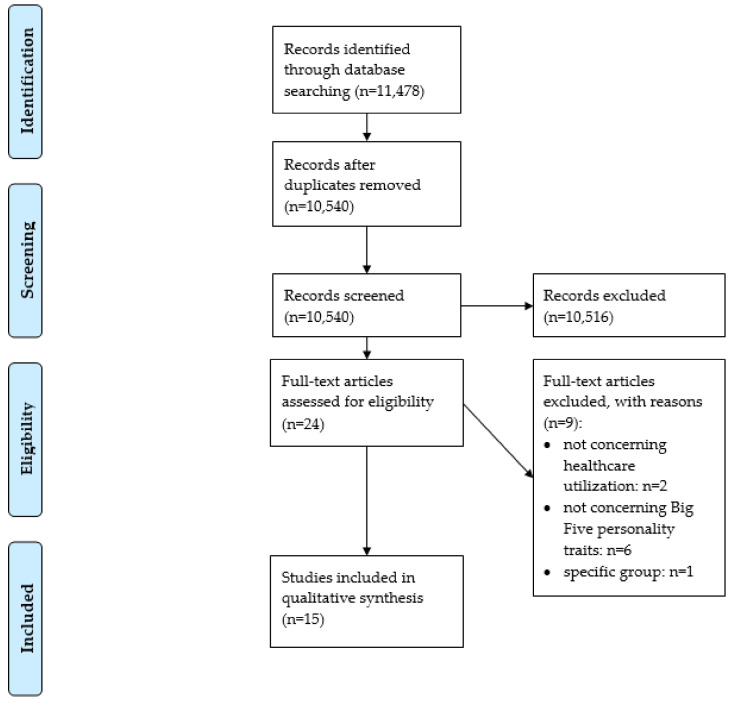
Preferred reporting items for systematic review and meta-analysis (PRISMA) flow diagram [13].

The study selection process is displayed in Figure 1 (flow chart) [13]. In total, n = 15 studies were included in the final synthesis (HCU: n = 14; COI (cost of illness): n = 1). An overview about the studies and main results are both presented in Table 2. If reported, adjusted results are presented in Table 2.

Data came from North America (n = 5, with: United States, n = 4; Canada, n = 1), and Europe (n = 10; with three studies from the Netherlands, two studies from Germany, two studies from the United Kingdom, one study from Denmark, one study from Sweden, and one study from Turkey). Ten cross-sectional and five longitudinal studies were identified. Three studies exclusively focused on neuroticism as a personality factor, whereas the other twelve studies used all five personality factors as explanatory variables. Several tools were used to assess the personality factors such as the 60-item version of the NEO-FFI (Five-Factor Inventory) or the short version of the Big Five inventory. With regard to HCU studies, there is a rather large variety in outcome measures. For example, a few studies focused on GP (general practitioner) or physician visits in general. Other studies have focused on the use of alternative or complementary medicine. Further studies have focused on other outcomes such as use of dental services. The single COI study focused on the economic costs of neuroticism.

Across the studies, the age ranged from 15 to 103 years. However, in the majority of the included studies, the average age was in the 40 s. While the proportion of women ranged from 0% to 100%, in more than half of the studies, the proportion of women was more than one-third and less than two-third. The sample size ranged from 69 to 37,185. Further details are given in Table 2.

In the following sections, we present our main findings for each personality factor separately: (1) extraversion and HCU, (2) agreeableness and HCU, (3) conscientiousness and HCU, (4) neuroticism and HCU or costs, as well as (5) openness to experience and HCU.

### 3.2. Extraversion and HCU

Twelve studies analyzed the association between extraversion and HCU. While one study found that a one sample deviation increase in extraversion increased the odds of emergency department use (OR (odds ratio) = 1.51, 95% CI (confidence interval): 1.03–2.21) [14], another study found that higher extraversion was associated with an increased risk of hospitalization (OR = 1.02, *p* < 0.05) [7]. Furthermore, it was found that extraversion was negatively associated with the use of any alternative medicine (OR = 0.65, 95% CI: 0.46–0.91) [15]. Moreover, it was found that extraversion was positively associated with the number of medical services (RR (relative risk) = 1.02, 95% CI: 1.02–1.02) [16].

### 3.3. Agreeableness and HCU

In total, n = 12 studies examined the link between agreeableness and HCU. Four out of these twelve studies found an association between agreeableness and HCU. More precisely, one study showed that higher agreeableness was associated with a higher use of complementary and alternative medicine (β = 0.21, *p* < 0.01) [17]. Another study found that a one sample deviation decrease in agreeableness was associated with increased odds of emergency department utilization (OR = 1.54, 95% CI: 1.05–2.22) [14]. Moreover, one study found a link between higher agreeableness and an increased number of medical services (RR = 1.02, 95% CI: 1.02–1.02) [16]. A further study found that higher agreeableness (β = 0.03, *p* < 0.05) was associated with a higher probability of any custodial nursing home use [6].

### 3.4. Conscientiousness and HCU

As in the case of agreeableness, twelve studies examined the link between conscientiousness and HCU. Out of these studies, two studies found an association. One study found an association between increased conscientiousness and an increased number of medical services (RR = 1.02, 95% CI: 1.02–1.02) [16]. In contrast, a second study found an association between lower conscientiousness and a higher probability of any custodial nursing home use (β = 0.02, *p* < 0.05) [6]. 

### 3.5. Neuroticism and HCU or Costs

All the fifteen included studies analyzed the link between neuroticism and HCU (n = 14) or costs (n = 1). Three studies found a link between neuroticism and an increased use of general practice/physician visits. More precisely, one study found that neuroticism was positively associated with physician visits (β = 0.01, *p* < 0.001) [7]. Another study found that neuroticism was positively associated with frequent attendance in general practice in women (*p* < 0.01) and men (*p* < 0.05) [18]. A link between higher neuroticism and increased primary care consultations was also found (year before baseline: ρ = 0.17, *p* < 0.001; year after baseline: ρ = 0.12, *p* = 0.003) [19].

Other studies have found a link between increased levels of neuroticism and the number of medical services (RR = 1.02, 95% CI: 1.02–1.02) [16], the use of any emergency department (β = 0.03, *p* < 0.001) [6], any custodial nursing home (β = 0.04, *p* < 0.05) [6], dental care use (neuroticism squared: β = −0.3, *p* = 0.03) [20], use of medication (upper quintile compared to the lower quintile: OR = 2.8, 95% CI: 1.8–4.5) [21], and increased likelihood of healthcare use (Mann–Whitney U test; *p* = 0.02) [22].

One study [23], examined the economic costs of neuroticism. This study [23] found that per capita excess equaled $12,362 per year (reference year was 2007) in individuals with the top 5% in terms of neuroticism (top 10%: $8243; top 25%: $5572). Total excess costs of neuroticism per 1 million individuals caused by the top 25% in terms of neuroticism ($1.393 billion) were about 2.5 times as high as the excess costs of other mental disorders.

### 3.6. Openness to Experience and HCU

Twelve studies investigated the association between openness to experience and HCU. Four out of these studies found an association. Two studies found an association between increased openness to experience and higher utilization of alternative medicine (OR = 1.65, 95% CI: 1.18–2.31) [15] or the use of complementary/alternative healthcare (*t*-test; *p* = 0.02) [24]. Another study revealed an association between increased openness to experience and an increased number of medical services (RR = 1.02, 95% CI: 1.02–1.02) [16]. Moreover, one study showed an association between higher openness to experience and a greater likelihood of any custodial home care use (β = 0.02, *p* < 0.05) [6].

### 3.7. Quality Assessment

In Table 3, the quality assessment of included studies is displayed. In total, the studies included fulfilled between 63% and 96% of the criteria. “Handling of missing data” (13.3%) and “performed sensitivity analysis” (40%) were the categories with the most unfulfilled criteria. The only COI study performed by Cuijpers [23], fulfilled nearly all of the criteria (96%)—except for the handling of missing data.

## 4. Discussion

The aim of our systematic review was to give an overview of empirical findings from observational studies examining the association between personality factors and HCU or costs.

In total, 15 studies were included in our systematic review. Out of these studies, a few studies point to an association between conscientiousness and HCU (with mixed evidence). Some more evidence was found for an association between higher agreeableness, higher extraversion, and higher openness to experience and increased HCU. The majority of studies analyzed found a link between higher neuroticism and increased HCU.

The positive association between extraversion and HCU, in particular hospitalization and emergency department (ED) use, appears very plausible to us because extraversion is associated with bad lifestyle habits [25,26]. Furthermore, perhaps more importantly, extraversion is positively associated with injury-prone behavior [27].

Some studies suggest a link between agreeableness and HCU. For example, the link between agreeableness and an increased use of complementary and alternative medicine appears plausible to us. For example, when a doctor recommends alternative or complementary medicine, a patient scoring high on agreeableness may tend to avoid conflicts with the doctor and may therefore have an increased use of complementary and alternative medicine.

Only two studies found an association between conscientiousness and HCU. This is somewhat surprising because conscientiousness is positively associated with health-promotion behavior [28] and preventive cancer screening [29], and negatively associated with accidents [30]. Future research is required to shed light on the underlying mechanisms.

The strong association between increased levels of neuroticism and increased HCU is one of the key findings of our systematic review. Most of the studies investigated found such a link. Other studies have also demonstrated that neuroticism is associated with increased use of mental health services [31,32]. Neuroticism is associated with experiences of negative emotions, which ultimately could affect HCU. Another study [7] speculated that this link could also be explained by poorer health behavior and worse coping with stress.

Openness to experience was also associated with HCU, particularly with an increased use of complementary/alternative medicine. Given the fact that individuals scoring high in openness to experience are open to different kinds of experiences (e.g., food, traveling abroad) [27], it appears to be plausible that these individuals are also open to the use of complementary/medicine.

There was some variety in the quality of the studies. For example, only a few studies performed sensitivity analysis. However, this is important to test the robustness of the findings and is recommended by current guidelines [33]. The handling of missing data was only described in two studies. However, the quality of studies included in our review was generally quite high. Furthermore, the quality tends to be somewhat higher in more recent studies. Therefore, we are quite confident that future studies may overcome these few shortcomings.

Some factors limit the comparability of the studies included. There was quite a large variety in measures used to assess personality factors. For example, while one study [21], used the Dutch Personality Inventory with 132 items, a recent study [24], used the Ten-Item Personality Inventory (10 items). Furthermore, there was a large variety in HCU domains—for example, from dental service use to use of alternative medicine to the frequency of GP visits. Most studies relied on self-reported HCU, which may result in some recall bias [34]. As far as data are available, future studies linking questionnaire data to claims data may be promising to overcome this potential limitation. The only existing cost study points to a considerable economic burden attributable to high levels of neuroticism. We strongly recommend future studies to confirm these findings. Moreover, future studies should also investigate the economic burden associated with the other four personality factors.

Some other factors are worth noting. There was a large heterogeneity in methods between the studies included. For example, while some studies only used bivariate analyses, other studies used panel regression models. Studies also differ in design (cross-sectional versus longitudinal), country, sample size, and participants (e.g., samples using primary care patients versus nationally representative samples). We could not detect any systematic differences that could explain our findings (for example, we could not detect that only studies from specific regions or solely high-quality studies found a link between neuroticism and HCU). In sum, we think that the results may simply reflect the fact that certain personality factors such as neuroticism are important for HCU in specific sectors. Nevertheless, future meta-analyses based on more homogenous studies (e.g., in terms of time horizon, outcome measures, or samples used) are required to support our current findings.

This systematic review has some strengths and limitations. Our current systematic review is the first synthesizing the evidence from observational studies regarding personality, HCU, and costs. A quality assessment was conducted. Focusing on observational data and not illness-specific samples can produce results that are widely generalizable. The steps of study selection, data extraction, and quality assessment were conducted using two independent reviewers. Due to study heterogeneity (e.g., outcome measures used), a meta-analysis could not be conducted. This is in line with Egger et al. [35], who recommended caution when performing a meta-analysis, particularly based on observational studies since it may lead to incorrect estimates for reasons of confounding and bias within the studies. However, it should be noted that recommendations for observational studies differ [36]. We focused on the widely acknowledged big five personality factors. However, future research is required to clarify whether other factors related to personality such as altruism, empathy, or locus of control [37] are associated with HCU or costs. For example, individuals who score high in altruism or empathy may have frequent doctor visits simply to avoid infecting others. Moreover, other models of personality structure exist such as the HEXACO model of personality. Future systematic reviews could also focus on these models.

## 5. Conclusions

Studies included in our systematic review suggest that personality factors, and particularly neuroticism, are associated with HCU. This knowledge is important for managing healthcare use. However, future research based on longitudinal data and studies investigating the link between personality characteristics and costs are required.

## Figures and Tables

**Table 1:** Search query (PubMed).

#	Search Term
#1	Personality [Title/Abstract]
#2	Big five [Title/Abstract]
#3	#1 OR #2
#4	Health care
#5	Health service *
#6	#4 OR #5
#7	Use
#8	Utili *
#9	#7 OR #8
#10	#6 AND #9
#11	cost
#12	Expense *
#13	Expenditure *
#14	Economic *
#15	#11 OR #12 OR #13 OR #14
#16	#10 OR #15
#17	#3 AND #16

Note: The asterisk (*) is a truncation symbol. The number sign (#) refers to the search order.

**Table 2:** Study overview and main findings.

First Author	Country	Assessment of Personality	Assessment of Healthcare Utilization	Study Type	Sample Description	Sample Size	Age	Proportion of Women (in %)	Results
Andersen (2012)	Denmark	Neuroticism: Mini International Personality Item Pool—Five-Factor Model measure (five items)	Visits to the general practitioner (duration: 1.5 years)	Cross-sectional	Representative (not specified) population	n = 5068	17–65	55.5%	Ordinal logistic regression revealed that people with neuroticism had more visits (OR = 1.2, 95% CI: 1.0–1.4) to the general practitioner.
							M = 46.1		
							SD = 12.9		
Chapman (2009)	United States of America	NEO Five-Factor Inventory (60 items)	Emergency department utilization (duration: three years)	Longitudinal	Recruited in primary care clinics	Baseline	65–94	63.8%	Generalized linear mixed models revealed that a one sample deviation * (which means 50th versus 83rd population percentile) increase in extroversion increased the odds of emergency department utilization by 51% (OR = 1.51, 95% CI: 1.03–2.21). An equal decrease in agreeableness (which means 50th versus 17th population percentile) increased the odds by 54% (OR = 1.54, 95% CI: 1.05–2.22).
						n = 747	M = 75.2		
							SD = 6.6		
Cuijpers (2010)	Netherlands	Neuroticism scale from the Amsterdam Biographic Inventory (14 items)	Costs: health service uptake in primary and secondary mental healthcare, out-of-pocket costs, and production losses	Cross-sectional	Netherlands Mental Health Survey and Incidence Study	n = 5504	18–65	49.1%	Total per capita excess costs were $12,362 per year (reference year: 2007) in the 5% highest scorers of neuroticism (top 10%: $8243; top 25%: $5572). Total excess costs of neuroticism per 1 million inhabitants resulting from the 25% highest scorers was $1.393 billion (top 10%: $824.3 million; top 5%: $618.1 million).
							M = 41.1		
							SD = 11.9		
den Boeft (2016)	Netherlands	NEO Five-Factor Inventory (60 items)	Trimbos and iMTA (instituut voor Medische Technology Assessment) questionnaire on costs associated with psychiatric illness	Longitudinal	Netherlands Study of Anxiety and Depression	Baseline	18–65	66.4%	Generalized estimating equations showed that all five personality traits (neuroticism: RR = 1.01, 95% CI: 1.01–1.02; extraversion: RR = 1.02, 95% CI: 1.02–1.02; openness: RR = 1.02, 95% CI: 1.02–1.02; agreeableness: RR = 1.02, 95% CI: 1.02–1.02; conscientiousness: RR = 1.02, 95% CI: 1.02–1.02) are significantly associated with healthcare use.
						n = 2981	M = 41.9		
							SD = 13.1		
Friedman (2013)	United States of America	NEO Five-Factor Inventory (number of items not specified)	Daily use of thirty different services (duration not specified)	Cross-sectional	Recruited for the Medicare Primary and Consumer-Directed Care Demonstration	n = 1074	65–100	72.7%	Controlling for various need variables from the Andersen Behavioral Model, neuroticism increased the use of any emergency department (β = 0.03, *p* < 0.001), and any custodial nursing home (β = 0.04, *p* < 0.05). Agreeableness (β = 0.03, *p* < 0.05) and conscientiousness (β = −0.05, *p* < 0.01) were associated with using any custodial nursing home as well. Openness to experience was associated with any custodial home care (β = 0.02, *p* < 0.05).
							M = 79.7		
							SD = 7.5		
Hajek (2017)	Germany	Short version of the Big Five Inventory (15 items)	Hospital stays for at least one night and number of physician visits (duration: three months)	Longitudinal	German Socioeconomic Panel	Baseline	17–103	54.3%	FE Poisson regressions showed that neuroticism was associated with physician visits (β = 0.01, p < 0.001). Furthermore, conditional FE logistic regressions showed that extraversion was associated with the risk of hospitalization (OR = 1.02, *p* < 0.05).
							M = 51.6		
						n = 37,185	SD = 16.7		
Honda (2005)	United States of America	Big five factor model (25 items)	Use of acupuncture, biofeedback, chiropractic, energy healing, exercise/movement therapy, herbal medicine, high-dose megavitamins, homeopathy, hypnosis, imagery techniques, massage, prayer/spiritual practice, relaxation/mediation, and special diet (duration: twelve months)	Cross-sectional	Midlife development in the United States Survey	n = 3032	25–74	50.2% among non-users of complementary and alternative medicine;	Logistic regression stated that openness (OR = 1.65, 95% CI: 1.18–2.31) and extraversion (OR = 0.65, 95% CI: 0.46–0.91) were associated with the use of any alternative medicine.
							(mean age and SD for the total sample not specified)	62.5% among users of complementary and alternative medicine	
Kennedy (1990)	United States of America	EPI-Q (Eysenck Personality Inventory Questionnaire) (18 items)	Dental utilization as measured by percent restored	Cross-sectional	VA (Veterans Affairs) Dental Longitudinal Study	n = 593	28–80	0.0%	A plot of neuroticism versus utilization stated that there was a curvilinear association: those scoring lowest and highest on this scale sought less treatment. Linear regressions showed that neuroticism squared was significantly associated with dental utilization (β = −0.3, *p* = 0.03).
							M = 47.8		
							SD = 8.1		
Metin (2019)	Turkey	Ten-Item Personality Inventory (10 items)	Holistic Complementary and Alternative Health Questionnaire	Cross-sectional	Academicians working for three leading universities in Turkey	n = 227	M = 38.9 SD = 10.4 (age range not specified)	65.6%	*t*-tests revealed that openness was positively associated with the use of complementary and alternative healthcare utilization (*p* = 0.02).
Reber (2018)	Germany	Short version of the Big Five Inventory (15 items)	Number of physician visits (duration: three months)	Longitudinal	German Socioeconomic Panel	n = 2140	In men:	31.0%	Poisson fixed effects regressions did not show any association for all big five personality domains and the number of physician visits.
							M = 48.3 years		
							SD = 9.4		
							In women:		
							M = 46.2		
							SD = 9.3		
							(age range not specified)		
Sirois (2008)	Canada	Big Five Factor Inventory (44 items)	Seven domains of complementary and alternative medicine (duration: one year)	Cross-sectional	Clients of complementary and alternative medicine	n = 184	15–86	83.2%	Hierarchical multiple regression revealed that agreeableness was associated with a higher use of complementary and alternative medicine (β = 0.21, *p* < 0.01).
							M = 41.4		
							SD = 13.7		
Tomenson (2012)	United Kingdom	Revised NEO Personality Inventory for neuroticism (number of items not specified)	Number of primary care consultations (duration: one year)	Cross-sectional	Random sample of the U.K. adult population	n = 961	25–65	54.0%	According to the Spearman correlation coefficient, there is a significant positive correlation between neuroticism and primary care consultations (year before baseline: ρ = 0.17, *p* < 0.001; year after baseline: ρ = 0.12, *p* = 0.003).
							M = 47.4		
							SD = 11.6		
van Hemert (1993)	Netherlands	Dutch Personality Inventory (132 items)	Using any medication daily (duration not specified)	Longitudinal	Data from the Epidemiological Preventive Investigation at Zoetermeer	n = 1167	45–64	100.0%	Controlling for age and education, logistic regressions showed that the upper quintile concerning neuroticism had higher chances than the lower quintile to use medication (OR = 2.8, 95% CI: 1.8–4.5).
							M = 53.2		
							SD = 5.7		
Wikehult (2005)	Sweden	Swedish universities Scales of Personality (91 items)	Receiving healthcare (duration: “currently”)	Cross-sectional	Victims of burn injury	n = 69	N = 46.1	23.2%	Mann–Whitney U tests stated a significant correlation between neuroticism and currently receiving healthcare (*p* = 0.022).
							SD = 15.5		
							(range not specified)		
Westhead (1985)	United Kingdom	Eysenck Personality Questionnaire (number of items not specified)	Being a frequent attender (ten percent most frequent attenders in each decade age group for each sex)	Cross-sectional	Practice population	n = 1491	Mean age, SD, and range not specified	50.9%	Chi-square tests revealed that mean scores for neuroticism were higher among frequent attenders, both among men (*p* < 0.05) and women (*p* < 0.01).

Notes: * With regard to the term “sample deviation”, Chapman [14], stated that “The NEO-FFI was scored using T scores (mean 50, sample deviation [SD] 10) according to national norms and scaled by normative SD units to provide meaningful interpretation. Thus, a 1-SD unit increase in each trait corresponded to the difference between the 50th population percentile to the 83rd, whereas a 1-SD decrease corresponded to the difference between the 50th population percentile to the 17th, shifts representing average to “high” and “low” levels, respectively, of a trait. Note that the sample standard deviations were comparable with those of the national norms (e.g., 10 T score points). The 50th percentile in the sample was half an SD lower than the national 50th percentile for neuroticism (e.g., T score of 45 rather than 50) and half an SD higher for agreeableness (e.g., T score of 55 rather than 50)”; Abbreviations in Table 2: OR = odds ratio; RR = relative risk; CI = confidence interval.

**Table 3:** Quality assessment.

**First Author (Year)**	**Type of Study (HCU/COI)**	**Study Objective**	**Inclusion and Exclusion Criteria**	**Cost Description**	**Comparison Group- or Disorder-Specific Costs**	**HCU Description**	**Comparison Group- or Disorder-Specific HCU**	**Currency**	**Reference Year**	**Perspective**	**Costs from More than One Category**	**Data Source**	**Valuation of Costs**	**Discounting**
Cuijpers (2010)	COI	✓	✓	✓	✓	n.a.	n.a.	✓	✓	✓	✓	✓	✓	✓
Andersen (2012)	HCU	✓	X	n.a.	n.a.	✓	✓	n.a.	n.a.	n.a.	n.a.	✓	n.a.	n.a.
Chapman (2009)	HCU	✓	✓	n.a.	n.a.	✓	✓	n.a.	n.a.	n.a.	n.a.	✓	n.a.	n.a.
den Boeft (2016)	HCU	✓	✓	n.a.	n.a.	✓	✓	n.a.	n.a.	n.a.	n.a.	✓	n.a.	n.a.
Friedman (2013)	HCU	✓	✓	n.a.	n.a.	✓	✓	n.a.	n.a.	n.a.	n.a.	✓	n.a.	n.a.
Hajek (2017)	HCU	✓	✓	n.a.	n.a.	✓	✓	n.a.	n.a.	n.a.	n.a.	✓	n.a.	n.a.
Honda (2005)	HCU	✓	✓	n.a.	n.a.	✓	✓	n.a.	n.a.	n.a.	n.a.	✓	n.a.	n.a.
Kennedy (1990)	HCU	✓	✓	n.a.	n.a.	✓	✓	n.a.	n.a.	n.a.	n.a.	✓	n.a.	n.a.
Metin (2019)	HCU	✓	✓	n.a.	n.a.	✓	✓	n.a.	n.a.	n.a.	n.a.	✓	n.a.	n.a.
Reber (2018)	HCU	✓	✓	n.a.	n.a.	✓	✓	n.a.	n.a.	n.a.	n.a.	✓	n.a.	n.a.
Sirois (2008)	HCU	✓	X	n.a.	n.a.	✓	✓	n.a.	n.a.	n.a.	n.a.	✓	n.a.	n.a.
Tomenson (2012)	HCU	✓	✓	n.a.	n.a.	✓	✓	n.a.	n.a.	n.a.	n.a.	✓	n.a.	n.a.
van Hemert (1993)	HCU	✓	✓	n.a.	n.a.	✓	✓	n.a.	n.a.	n.a.	n.a.	✓	n.a.	n.a.
Westhead (1985)	HCU	✓	✓	n.a.	n.a.	✓	✓	n.a.	n.a.	n.a.	n.a.	✓	n.a.	n.a.
Wikehult (2005)	HCU	✓	✓	n.a.	n.a.	✓	✓	n.a.	n.a.	n.a.	n.a.	✓	n.a.	n.a.
% of criteria fulfilled by studies		100	86.7	100	100	100	100	100	100	100	100	100	100	100
**First Author (Year)**	**Missing Data**	**Statistics**	**Consideration of Confounders**	**Sensitivity Analysis**	**Sample Size (Sub-group)**	**Demographics**	**Arithmetic Mean Costs**	**SD (SE) or CI**	**Results Discussed with Respect to Other Studies**	**Results Discussed Regarding Generalizability**	**Limitations**	**Conclusion Supported by Data**	**Conflict of interest/funders**	**% of Criteria Fulfilled by Study**
Cuijpers (2010)	X	✓	✓	✓	✓	✓	✓	✓	✓	✓	✓	✓	✓	95.8
Andersen (2012)	X	✓	✓	✓	✓	✓	n.a.	n.a.	✓	✓	✓	✓	✓	87.5
Chapman (2009)	✓	✓	✓	X	✓	✓	n.a.	n.a.	✓	✓	✓	✓	✓	93.8
den Boeft (2016)	X	✓	✓	X	✓	✓	n.a.	n.a.	✓	✓	✓	✓	✓	87.5
Friedman (2013)	X	✓	✓	✓	✓	✓	n.a.	n.a.	✓	✓	✓	✓	✓	93.8
Hajek (2017)	X	✓	✓	✓	✓	✓	n.a.	n.a.	✓	✓	✓	✓	✓	93.8
Honda (2005)	X	✓	✓	X	✓	✓	n.a.	n.a.	✓	✓	✓	✓	✓	87.5
Kennedy (1990)	X	✓	✓	X	✓	✓	n.a.	n.a.	✓	X	✓	✓	✓	81.3
Metin (2019)	X	✓	X	X	✓	✓	n.a.	n.a.	✓	✓	✓	✓	✓	81.3
Reber (2018)	X	✓	✓	✓	✓	✓	n.a.	n.a.	✓	✓	✓	✓	✓	93.8
Sirois (2008)	✓	✓	✓	✓	✓	✓	n.a.	n.a.	✓	✓	✓	✓	✓	93.8
Tomenson (2012)	X	✓	✓	X	✓	✓	n.a.	n.a.	✓	✓	✓	✓	✓	87.5
van Hemert (1993)	X	✓	✓	X	✓	✓	n.a.	n.a.	✓	X	✓	✓	✓	81.3
Westhead (1985)	X	✓	✓	X	✓	X	n.a.	n.a.	✓	X	X	✓	X	62.5
Wikehult (2005)	X	✓	✓	X	✓	✓	n.a.	n.a.	✓	✓	X	✓	✓	81.3
% of criteria fulfilled by studies	13.3	100	93.3	40	100	93.3	100	100	100	80	86.7	100	93.3	

Notes: HCU: healthcare use; n.a.: not applicable; ✓: quality criterion was fulfilled; X: quality criterion was not fulfilled.
